# Evaluating the effectiveness of the Play Active policy intervention and implementation support in early childhood education and care: a pragmatic cluster randomised trial protocol

**DOI:** 10.1186/s12889-022-12729-5

**Published:** 2022-02-14

**Authors:** Andrea Nathan, Emma Adams, Stewart Trost, Donna Cross, Jasper Schipperijn, Matthew McLaughlin, Ashleigh Thornton, Georgina Trapp, Leanne Lester, Phoebe George, Elizabeth Wenden, Hayley Christian

**Affiliations:** 1 Telethon Kids Institute, Perth, Australia; 2 School of Human Movement and Nutrition Sciences, University of Queensland, Brisbane, Australia; 3 Centre for Child Health Research, The University of Western Australia, Perth, Australia; 4 Department of Sports Science Clinical Biomechanics, University of Southern Denmark, Odense, Denmark; 5 School of Population and Global Health, The University of Western Australia, Perth, Australia; 6 School of Human Sciences, The University of Western Australia, Perth, Australia

**Keywords:** Physical activity, Screen time, Sedentary, Childhood, Childcare, Intervention, Policy, Implementation

## Abstract

**Background:**

Daily physical activity is critical during the early years of life for facilitating children’s health and development. A large proportion of preschool children do not achieve the recommended 3 h of daily physical activity. Early childhood education and care (ECEC) services are a key setting to intervene to increase physical activity. There is a significant need for ECEC specific physical activity policy, including clearer guidelines on the amount of physical activity children should do during care, and strategies for implementation of these guidelines.

**Methods:**

This study is a pragmatic cluster randomised trial to evaluate the effectiveness of the Play Active physical activity policy intervention to improve early childhood education and care educator’s physical activity-related practices. The central component of Play Active is an evidence-informed physical activity policy template which includes 25 practices to support nine age-specific recommendations on the amount of physical activity and sedentary time, including screen time, young children should do while in care. There are six implementation support strategies to facilitate physical activity policy implementation: (i) personalise policy (services select at least five of the 25 practices to focus on initially); (ii) policy review and approval; (iii) a resource guide; (iv) a brief assessment tool for monitoring children’s energetic play; (v) professional development; and (vi) Project Officer implementation support (phone calls). A total of 60 early childhood education and care services will be recruited from metropolitan Perth, Western Australia. After baseline assessment, services will be randomly allocated to either intervention or wait-listed comparison conditions. Primary (educator-reported frequency and amount of daily time provided for children’s physical activity, sedentary and screen time) and secondary (educator physical activity-related practices, self-efficacy, motivation, attitudes and beliefs, social support, and supportive physical environment) outcomes will be assessed at baseline and post-intervention, after intervention services have had a minimum 3 months of policy implementation within their service.

**Discussion:**

The Play Active trial will rigorously evaluate a novel physical activity policy intervention with implementation support that promotes positive physical activity behaviours in educators and children attending ECEC. If effective, the program could be adapted, scaled-up and delivered in ECEC services nationally.

**Trial registration:**

Australian New Zealand Clinical Trials Registry ACTRN12620001206910 (date of registration 13/11/2020).

**Supplementary Information:**

The online version contains supplementary material available at 10.1186/s12889-022-12729-5.

## Background

Daily physical activity is critical during the early years of life [1]. Regular physical activity provides children with health and developmental benefits, including a healthy weight, improved bone health, cardiovascular fitness, and enhanced cognitive, emotional and psychosocial development [2]. A large proportion of preschool children do not achieve the recommended 3 h of daily physical activity [3–5]. A key setting of influence for young children’s physical activity is centre-based childcare services, referred to as early childhood education and care (ECEC) services in Australia. These include preschools, long-day care services and kindergartens that provide educational and developmental activities for children prior to the commencement of compulsory schooling [6]. Research shows that ECEC services have a larger influence on variations in levels of young children’s (2–5 years) physical activity than socio-demographic factors [7]. Yet systematic reviews of intervention studies implementing physical activity policies, practices or programmes within ECEC services indicate little evidence of benefit on young children’s physical activity [6]. This is due to lack of efficacy that the intervention improves children’s physical activity, lack of implementation supports (e.g., management support, external resources, training), implementation barriers (e.g., lack of support, negative attitudes, poor self-efficacy, lack of physical space), the intervention not being conducive to real-word conditions (loss of effect size) and the insufficient use of evidence-based behaviour change frameworks [6].

There is a need for multilevel and multicomponent implementation interventions in ECEC settings that are underpinned by comprehensive evidence-based behaviour change frameworks [8]. Social-ecological models of behaviour change integrate many behaviour change theories [9]. Four core concepts underpin social-ecological models: 1) multiple levels of factors influence health behaviours from individual factors through to a wide range of social and environmental factors; 2) complex and dynamic interactions of multilevel factors work together to influence behaviour; 3) social-ecological models are most useful when tailored to specific health behaviours; and 4) interventions systematically targeting multilevel factors of influence are most effective in changing and sustaining behaviours at the population level [10–12]. Physical activity intervention research based upon a social-ecological framework may lead to more effective implementation and enable successful scale up [10].

Consistent with socio-ecological models, implementation of physical activity policy is important for changing the physical activity practices of educators to positively impact children’s physical activity levels in care and over the longer term. In ECEC settings, the policy environment can influence children’s physical activity in varying ways. This can be indirectly through having a written ‘health and safety’ policy that protects children from extreme weather conditions when playing outdoors (i.e., sun protection), or a specific written physical activity policy [13], through to regulatory requirements and standards requiring services to have and implement a physical activity policy [14, 15]. However, only half of all Australian, New Zealand, Canadian and US services have a written physical activity policy [16–19] with considerable within country variation [16, 20]. Interventions to develop and effectively implement written physical activity policies are needed in ECEC [13, 21].

In addition to ECEC services having policies with clear guidance on the amount and type of physical activity children should do whilst attending care, strategies to support the implementation of physical activity policy-related practices are also needed. A number of key physical activity practices have been identified as necessary for successful policy implementation, including practices related to management, supervisors and educators [22]; the ECEC physical environment; communicating with families; and accreditation, monitoring and review [13]. As educators are recognised as gatekeepers of children’s behaviour in ECEC, improving educator physical activity practices is an important component of effective physical activity policy implementation [23].

While educators agree on the importance of physical activity for children’s health and development, there appears to be low awareness of the amount, type and nature of physical activity that is needed for it to be beneficial for children’s health and wellbeing [13]. Moreover, most Australian educators do not receive professional development and training in relation to physical activity [13]. Pilot studies have shown outdoor nature-based play and fundamental movement skill professional development programs tailored to the ECEC setting to be effective in improving educators’ self-efficacy to engage children to be physically active [24]. Further research is needed to identify the most effective content and means of delivering quality professional development and training for educators, to provide them with the necessary skills and tools for promoting the physical activity levels of children in their care [8].

To address these needs, the Play Active program was developed. Play Active is a physical activity policy intervention with accompanying implementation support strategies, with the overarching goal to increase physical activity levels in young children (< 5 years) attending ECEC. The central component of Play Active is an evidence-informed physical activity policy template containing 25 practices to support nine key age-specific recommendations and two key statements which provide clear guidance on the amount of physical activity and sedentary time, including screen time, young children should do while attending ECEC [13]. There are six implementation support strategies to facilitate physical activity policy implementation within ECEC services:

(i) Personalise policy: services select at least five from 25 practices within the physical activity policy template to focus on initially [13].
(ii) Policy review and approval: services submit draft policy to a Play Active Project Officer; two Project Officers subsequently review the policy against minimum criteria and approve or provide tailored feedback to reach approval.
(iii) Resource guide: with practical tips and activity suggestions, resources are mapped to the 25 practices within the policy.
(iv) Brief assessment tool: for monitoring young children’s physical activity levels, provided within the resource guide.
(v) Professional development: training on fundamental movement skills and active play-based learning, provided by Play Active partners.
(vi) Project Officer implementation support: including both weekly follow-up (phone and/or email) to complete the policy review and a mid-implementation prompt (phone call) to determine whether policy implementation has commenced.

This paper describes the protocol of a pragmatic cluster randomised trial to evaluate the effectiveness of the Play Active physical activity policy intervention (with implementation support strategies) on increasing the frequency and amount of time each day ECEC educators provide for young children’s physical activity, sedentary and screen time (primary aim). Secondary aims will examine the effects of the intervention on:

1. Educator physical activity practices, specifically increasing modelling of physical activity, programming and planning physical activity, and promoting and encouraging physical activity, and decreasing use of physical activity for managing misbehaviour.
2. Increasing the proportion of ECEC educators who report a positive attitude, feel confident and motivated, feel supported by management and co-workers, and have a supportive physical space for promoting young children’s physical activity.
3. The proportion of ECEC services with staff who take up and complete physical activity-related professional development and training.

The protocol follows the SPIRIT guidelines (Additional file 1) [25].

## Methods

### Study design and setting

This pragmatic cluster randomised trial design involves 60 ECEC services in Perth metropolitan and Peel regions of Western Australia. The design was selected to maximise the applicability to ECEC services, that is, to test the Play Active physical activity policy intervention and accompanying implementation support strategies with a wide range of ECEC staff (directors and educators), while simultaneously enabling the program to be as close to real world conditions as possible [26]. At the conclusion of baseline data collection, ECEC services will be randomly allocated to either the intervention (physical activity policy template plus six implementation support strategies) or wait-listed comparison groups. Primary and secondary trial outcomes will be assessed for change immediately following the (up to 6 month) intervention period. The trial is registered through the Australian New Zealand Clinical Trials Registry (reference number 12620001206910, registered 13/11/2020). An overview of the study design, schedule for enrolment and study assessments is shown in Table 1.

**Table 1:**
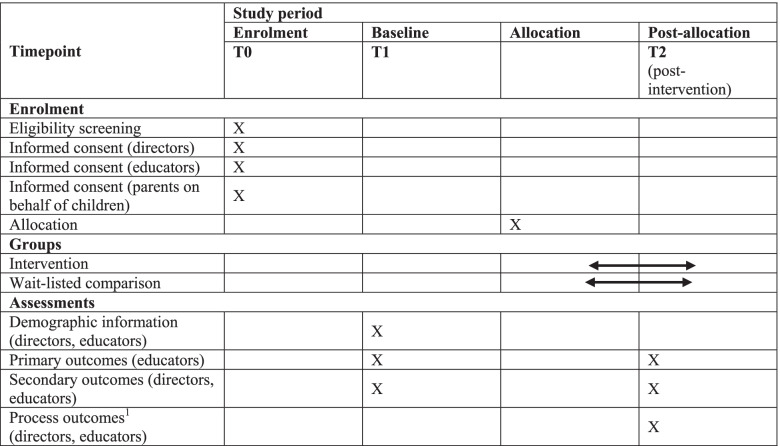
Schedule of enrolment, intervention, and assessments for the evaluation of the Play Active policy intervention and implementation support

Primary outcomes: educator-reported frequency and amount of time per day provided for children’s physical activity, sedentary and screen time. Secondary outcomes: educator physical activity practices, self-efficacy, motivation, attitudes and beliefs, social support, supportive physical environment and professional development training. ^1^For services allocated to intervention group only

The setting for this pragmatic trial are ECEC long-day care services, which provide centre-based childcare by professional staff for large groups of young children prior to formal compulsory schooling [27]. In Australia, ECEC long-day care services are mostly privately owned by providers, with the vast majority of providers approved to operate a single service only (81%), requiring enrolment and a daily fee for service [28]. ECEC services are highly regulated; the Australian National Quality Framework for ECEC outlines minimum standards across seven quality areas, including quality area 2.1.3 which states ‘healthy eating and physical activity are promoted and appropriate for each child’ [15]. As of February 2021, the regulating body, the Australian Children’s Education and Care Quality Authority reported there were 557 ECEC long-day care services located in the Perth and Peel metropolitan region of Western Australia. As the National Quality Framework also sets out minimum educator to child ratio requirements for ECEC services, there was an average of 66 approved places for children per service in the study region (range = 13–223 children).

### Eligibility criteria

Long-day ECEC services located in Perth and Peel, Western Australia will be eligible to participate. Ineligibility criteria include: long-day ECEC services catering exclusively for children requiring specialist care, mobile preschools, and Department of Education and Communities preschools; ECEC services already involved in an alternative randomised controlled trials currently underway in Perth, Western Australia; and ECEC services that have had a significant change in management within the last 3 months or expect a change in management in the next 3 months. Within each ECEC service, all permanent part- and full-time staff educators will be eligible to participate in the trial. Casually employed educators will be excluded. Within each ECEC service, all attending children aged 1–5 years who have not yet commenced formal schooling (pre-primary) will be eligible for participation in the study with parental consent. Exclusion criteria for attending children are a recognised disability (physical, emotional/behavioural or intellectual) that would affect participation in physical activity, and attendance of full-time formal schooling which would prohibit whole days spent in ECEC.

### Participant recruitment and consent

To closely align with real-world dissemination strategies, ECEC services will be recruited via an ‘Expression of Interest’ form available on one of the study partner’s (Cancer Council Western Australia) website. A launch event was used to publicise the new Play Active program and inform services how to express their interest in receiving the physical activity policy template and implementation support strategies. ECEC services who express interest will be contacted by the research team using a combination of modes (e.g., email, telephone) to invite them to take part in the pragmatic trial. ECEC providers with multiple services will be contacted and all services invited to participate. Recruitment commenced January 2021 and is ongoing. Eligible service directors will be provided with study information and asked to provide consent for their service to participate in the trial. Directors will then be asked to provide contact details for their eligible educators so that the research team can invite them to participate (consent process included as part of the educator survey). Directors will also be provided with parent information packs to distribute to their families and consent forms for children’s data from the educator completed brief energetic play assessment tool to be provided to the research team.

### Sample size and power

Based on a 15 min per day change in the primary outcome variable (i.e., average change in educator reported time allocated for children to be physically active indoors and outdoors), this trial will require a total of 60 services with 108 educators per group (80% power, 2-tailed alpha level of 0.05) to detect a moderate effect size of 0.4. After adjusting for clustering of educators within services, an educator ICC of 0.01–0.05 and a 30% dropout at follow-up data collection, 309 educators (6 per service) from 60 services will be recruited. This represents 11% of long-day care services in the Perth, Western Australia region.

### Randomisation

Services will be randomised to the intervention or wait-listed comparison group after baseline data collection. Using a central randomisation procedure, the randomisation sequence will be generated using a computerised random number function in Microsoft Excel. Due to the increased chances of contamination between services of the same provider if allocated to different groups, these services will be randomly allocated to the same group or two different groups grouped by geographical location. The research team member generating the allocation sequence and assigning services to their group will not be involved with recruitment, data collection or intervention delivery. Due to the nature of the intervention (i.e., implementation of a policy), it is impossible to blind services to their group allocation, thus this will be an open trial without masking and services will be aware of the group they are assigned to.

### Play Active program for ECEC

The Play Active program consists of an evidence-informed editable physical activity policy template containing nine recommendations, two key statements and 25 practices and six policy implementation support strategies.

#### Development of the program

Play Active was developed in consultation and co-produced [29] with formal and informal study partners including representatives from ECEC service providers, ECEC professional associations, government (health; local government, sport and cultural industries) and non-government organisations (National Heart Foundation, Cancer Council Western Australia, Nature Play Australia) all focused on promoting children’s physical activity. Educators have provided critical input to the Play Active program development and implementation support strategies through prior formative qualitative research. Parents provided input to the Play Active program development, implementation and evaluation via a Consumer Reference Group which meets quarterly.

The physical activity policy template was developed using a modified three round Delphi methodology; full details of the methods have been published previously [13]. The policy template aligns with national and international 24-h movement guidelines and provides age-specific guidelines on the amounts of physical activity and sedentary time, including screen time, young children should do while attending ECEC. The policy template includes 25 educator-related physical activity practices which were identified through focus groups with educators and the Delphi process.

#### Physical activity policy template

ECEC directors will be provided via email with an editable version (word document) of the physical activity policy template, which includes:

- Two key statements which apply to all children and nine recommendations that are age-specific (see Table 2 for details)
- 25 practices (known as procedures in the ECEC context) outline specific opportunities to achieve the recommendations and are specific to management and educators (14 practices), the physical environment (four practices), parent and carer engagement (five practices), and policy monitoring and review (two practices) [13].

**Table 2:** Key statements and recommendations in the Play Active policy template [13]

Key statement	Recommendation	Age group		
		Infants (under 1 year)	Toddlers (1–2 years)	Kindergarten (3–5 years)
Encourage physical activity in young children	To meet the Australian 24-h movement guidelines for the early years, provide children with at least 180 min of physical activity daily, via a variety of physical activities spread throughout the day. More is better	˟	✓	✓
	For kindergarten children, this will include at least 30 min of energetic play each day at ECEC. More is better	˟	˟	✓
	For infants who are mobile provide physical activity in a variety of ways, mainly through supervised, interactive floor-based play such as crawling and games. More is better	✓	˟	˟
	For infants not yet mobile, provide at least 30 min of tummy time spread throughout the day, including reaching and grasping, pushing and pulling	✓	˟	˟
Limit sedentary behaviours in young children	Toddlers and kindergarten children will not be confined for more than 60 min at a time (e.g., in a stroller or highchair). Children will not sit for extended periods (expect when engaged with a caregiver in an activity, e.g., reading and storytelling). Less is better	˟	✓	✓
	Sedentary screen time for purposes other than learning will not be allowed	˟	✓	✓
	Screen time for infants is NOT recommended	✓	˟	˟
	Ensure cots, car seats, and highchairs are used for their primary purpose only (cots for sleeping, car seats for vehicle travel, and highchairs for eating)	✓	˟	˟
	Limit the use of equipment such as strollers, swings, and bouncer seats/chairs for holding infants while they are awake	✓	˟	˟

#### Policy implementation supports

Alongside the physical activity policy template, directors will be provided with six implementation support strategies. The itemised components of each implementation support strategy are detailed in Additional file 2 and summarised below.

(i) Personalise policy: ECEC service staff will be encouraged to personalise the physical activity policy template to suit their service and the needs of their attending children and families. Services are asked to *select at least five from 25 practices* to prioritise during initial implementation. To support ECEC services in this, seven high impact and low effort practices, based on previous testing [13] are indicated.
(ii) Policy review and approval: Services are asked to submit their draft physical activity policy (based on the template) to Play Active. Two Play Active Project Officers independently review the policy and approve policies that meet minimum criteria. The minimum requirements for physical activity policies to be approved are that it include: two (out of two) key statements; nine (out of nine) recommendations; and at least five (out of 25) practices to prioritise implementing during the trial’s intervention period. After service’s physical activity policies have been approved as meeting the minimum requirements, services will be provided with the remaining implementation support strategies to assist with policy implementation and encouraged to implement the policy over a minimum period of 3 months. Services will be granted a period of up to 2 months for personalising and submitting physical activity policies for approval. After 2 months, any services that do not submit a physical activity policy for review and approval will be provided with all policy implementation supports. Personalisation of the policy and the policy review process is important to enhance policy ownership and implementation of the policy within each service. This process aligns with standard practice in ECEC service’s when initiating and implementing any new policy.
(iii) Resource guide: An evidence-informed resource guide (hard copy and digital PDF) with practical tips describing how to implement each of the 25 physical activity practices into daily practice has been developed. The resource guide consists of the following sections: glossary of terms; policy template; benefits of being an accredited Play Active service; and a section covering each of the 25 practices. The 25 practices are consistent with the policy template and outline practical strategies educators can use, an evidence-informed explanation of what it means, and various evidence-based helpful resources for more information. One physical copy of the resource guide is provided to each service as well as an electronic copy.
(iv) Brief assessment tool: A brief monitoring tool has been created for educators to monitor children’s physically activity whilst attending ECEC. The Energetic Play Assessment Tool was adapted from a brief instrument developed by Rice and colleagues [30]. For each child, educators record how much of a typical day in the last month was spent energetically playing (e.g., running, jumping, skipping, dancing, riding, climbing and energetic games) indoors and outdoors. There are five response options (very rarely energetic, rarely energetic, sometimes energetic, often energetic, very often energetic) recorded across four parts of the day (arrival to morning tea, morning tea to lunch, lunch to afternoon tea, afternoon tea to departure). Reliability and validity of the tool is currently being assessed. The tool is included as part of the resource guide.
(v) Professional development: Training to upskill educators in providing more physical activity opportunities for children in their care, including specific skills on developing fundamental movement skills and active play-based learning. The training for educators will be made available by study partners (Nature Play WA and KIDDO). The Nature Play WA training involves five self-paced centre-based e-learning modules with associated resources. Each module has up to 40 segments of content, equates to 3–4 h of training per module and is expected to take at least 6 weeks to be completed. As part of the trial there is a cost of $99 per module per service. The module topics include: the importance of being a playful educator; active and playful outdoor learning environments; using outdoor play to increase physical activity; planning cycles for active outdoor play; and documenting as a process of reflection, intention and communication. The KIDDO professional development training will be provided to services for free as part of the trial and focusses on developing children’s physical literacy (fundamental movement skills). It involves six, 30–60-min online training modules which individual educators complete at their own pace. Module topics cover the importance of physical literacy; what are fundamental movement skills; teaching fundamental movement skills in early childhood; active ECEC environments; physical literacy in ECEC; and promoting motivation in children. The research team has shown in pilot studies that both professional development training options are feasible and effective in improving educators’ self-efficacy to engage children to be physically active in ECEC [24].
(vi) Project Officer implementation support: including both weekly follow-up (phone and/or email) to complete the policy review and a mid-implementation prompt (phone call) to determine whether policy implementation has commenced. Service directors will be provided weekly email and phone call reminders to return their policy for review for up to 2 months.

### Wait-listed comparison

Services allocated to the wait-listed comparison group will be instructed to continue their usual practices around physical activity for the duration of the trial. After the conclusion of the post-intervention data collection, wait-listed comparison services will be provided with a copy of the Play Active physical activity policy template and the six policy implementation supports.

### Participant characteristics

Demographic information will be collected from directors and educators at baseline and will include age, gender, education, position title, months/years of experience working in current ECEC service, and months/years of experience working in ECEC sector. In addition, educators will be asked the name of the room they work in, the age/s of children in this room, and the number of hours per week usually spent working in this room.

### Primary outcomes

Change in educator physical activity practices related to the frequency or amount of time provided each day for physical activity, sedentary and screen time of young children in care will be assessed using established items drawn from existing validated instruments (e.g., Nutrition and Physical Activity Self-Assessment for Child Care (NAPSACC) [31] and Environment and Policy Assessment and Observation (EPAO) – Self Report tool [32]), which have been modified for the Australian ECEC context [27] and found to have acceptable test-retest reliability [33]. The measures align with the nine recommendations outlined in the editable policy template (see Table 3 for detail) and include educator-reported time provided each day for outdoor physical activity, indoor physical activity, educator-led physical activity, energetic play, tummy time (infants only), sitting time, screen time and daily frequency of outdoor play time.

**Table 3:** Primary outcome measures corresponding to Play Active policy template recommendations

Recommendation	Educator survey items^a^	Response option
To meet the Australian 24-h movement guidelines for the early years, provide children with at least 180 min of physical activity daily, via a variety of physical activities spread throughout the day. More is better	The amount of time I provide children for indoor physical activity each day is	Seven categories: < 30 min; 30–59 min; 60–89 min; 90–119 min; 120–149 min; 150–179 min; ≥180 min
	The amount of time I provide children for outdoor physical activity each day is	Seven categories: < 30 min; 30–59 min; 60–89 min; 90–119 min; 120–149 min; 150–179 min; ≥180 min
	I provide children with outdoor play time	Six categories: 0 times per day; 1 time per day; 2 times per day; 3 times per day; 4 times per day; 5+ times per day
	The amount of educator led physical activity I provide children each day is	Seven categories: < 30 min; 30–59 min; 60–89 min; 90–119 min; 120–149 min; 150–179 min; ≥180 min
For kindergarten children, this will include at least 30 min of energetic play each day at ECEC. More is better	The amount of time I provide to children for energetic play each day is	Five categories: < 15 min; 15–29 min; 30–44 min; 45–59 min; ≥60 min
For infants who are mobile provide physical activity in a variety of ways, mainly through supervised, interactive floor-based play such as crawling and games. More is better	I provide infants and babies with supervised interactive floor-based play, including crawling and games	Seven categories: Never; rarely; sometimes; often; very often; always; not applicable
For infants not yet mobile, provide at least 30 min of tummy time spread throughout the day, including reaching and grasping, pushing and pulling	I provide non-crawling infants with tummy time each day for a total of	Six categories: < 15 min; 15–29 min; 30–44 min; 45–59 min; ≥60 min; not applicable
Toddlers and kindergarten children will not be confined for more than 60 min at a time (e.g., in a stroller or high chair). Children will not sit for extended periods (expect when engaged with a caregiver in an activity, e.g., reading and storytelling). Less is better	Outside of sleep and meal times, the longest children in my care are expected to remain seated at any one time is	Seven categories: < 10 min; 10–19 min; 20–29 min; 30–39 min; 40–49 min; 50–59 min; ≥60 min
Sedentary screen time for purposes other than learning will not be allowed	The amount of screen time I provide each day to children is	Six categories: 0 min; 1–14 min; 15–29 min; 30–44 min; 45–59 min; ≥60 min
Screen time for infants is not recommended		
Ensure cots, car seats, and high chairs are used for their primary purpose only (cots for sleeping, car seats for vehicle travel, and high chairs for eating).	I use cots, car seats and high chairs for their primary purpose only (cots for sleeping, car seats for vehicle travel, and high chairs for eating)	Six categories: Never; rarely; sometimes; often; very often; always
Limit the use of equipment such as strollers, swings, and bouncer seats/chairs for holding infants while they are awake	I use strollers, swings and bouncer chairs to hold infants while they are awake	Seven categories: Never; rarely; sometimes; often; very often; always; not applicable

^a^Items align with nine recommendations in the ECEC physical activity policy template and are based upon existing items [31–33]

### Secondary outcomes

Secondary outcomes will include changes in educator physical activity practices related to role modelling physical activity (e,g., I join children in physically active play), programming and planning physical activity (e.g., I incorporate physical activity into room routines and transitions), promoting and encouraging physical activity (e.g., I talk with children about the importance of physical activity), and not using physical activity for managing misbehaviour (e.g., I take away five or more minutes of active play time if children misbehave). These practices will be assessed with a total of 30 items sourced from the NAPSACC instrument, EPAO instrument or developed specifically for purposes of this trial to align with the practices outlined in the physical activity policy template [31–33]. All items are assessed on a six-point Likert scales from Never (1) to Always (6) in the educator survey administered at baseline and post-intervention.

Secondary outcomes will also include changes in educator self-efficacy (eight items; e.g., I feel able to provide children with opportunities for energetic play throughout the day) and motivation (eight items; e.g., I am motivated to provide children with opportunities for energetic play throughout the day), with items aligned specifically with the Play Active policy template recommendations. Changes in educators attitudes and beliefs towards children’s physical activity and sedentary behaviour will be assessed using nine items developed to be fit-for-purpose based on the key barriers and enablers for implementing a physical activity policy in ECEC identified through the Delphi process [13]. Changes in educator-perceived social support provided by management and co-workers for encouraging physical activity and limiting sedentary behaviours will be assessed with four items. The above items are all context-specific to the Play Active program and consistent with standard types of physical activity items measured on seven-point Likert scales from Strongly Disagree (1) to Strongly Agree (7) [34–36].

Service directors will be surveyed at baseline and post-intervention to collect information on changes in the degree to which the ECEC physical environment supports young children’s physical activity using established items from the EPAO’s space, equipment and environment subscale [32], which has been modified for the Australian ECEC context and shown to provide reliable measures [33].

Given the short three-month policy implementation period for this trial, it is unlikely there will be a significant change in children’s physical activity. However, to ensure we capture any potential changes, data will be obtained from the Energetic Play Assessment Tool, which will be used both as an implementation support strategy and a data collection tool.

### Process outcomes

To understand the mechanisms by which policy implementation support strategies did or didn’t work, process evaluation outcomes will be measured post-intervention for intervention service directors and educators [37, 38]. Survey measures will include the reach of the implementation support strategies, the acceptability and appropriateness of the Play Active program (policy and implementation support strategies) and educator feedback on adaptations to improve the program.

Uptake of the implementation support strategies (reach) will be measured using multiple methods. These include project management logs to see if the policy template is returned for review; website data from partners (Nature Play WA and KIDDO) to assess the uptake of professional development by educators as well as self-report items in the educator post-intervention survey. To further assess reach, educators will also be asked whether their service has a physical activity policy and how often they used the resource guide (assessed on a seven-point Likert scale from once (1) to more than once per day (7)). To assess knowledge of the Play Active program, educators will also be asked a series of true/false knowledge questions based on the content of the physical activity policy recommendations (coverage).

Director- and educator-reported satisfaction (acceptability) with the Play Active program, including open ended questions about what they liked most and what could be improved will be collected. The educator post-intervention survey will also include items on the usefulness (appropriateness) of the resource guide and professional development (assessed on a five-point Likert scale from not at all useful (1) to extremely useful (5)), and what they liked most (acceptability) and what could be improved (suggested adaptations).

### Participant response and retention

All services will take part in baseline and post-intervention data collection according to the schedule in Table 1. Director and educator surveys will be administered online using the secure REDCap survey platform [39, 40]. Survey links will be emailed to all service directors and educators with reminders sent via the REDCap platform, email and telephone. Service directors will be contacted weekly via phone or email to remind them to complete their survey and to encourage their educators to complete their survey. Hard copy and PDF director and educator surveys will also be made available to assist with survey completion. Baseline and post-intervention educator surveys will be linked through the REDCap platform via unique participant codes, so that matching of repeat measures is automatic. Any data resulting from hard or soft copy surveys will be double entered into a Microsoft Excel spreadsheet by two different research team members. A third team member will confirm discrepancies and amend as required. Participant data will be managed on a secured electronic database (REDCap) and hard-copy forms stored securely at the research facility.

Well-established retention strategies such as contacts via email, phone calls and face to face visits will be used to thank services for their participation to date and remind them to complete all components of data collection so as to minimise attrition at post-intervention data collection.

### Statistical analysis

Analysis will be undertaken following intention-to-treat principles. To examine the representativeness of the study sample, service-level characteristics will be compared with the full sampling frame of Perth and Peel ECEC services at the time of recruitment using data available from Australian Children’s Education and Care Quality Authority [28]. The primary outcome variables of educator-reported frequency and time provided for children to be physically active will be analysed using generalised linear mixed effects models and will include fixed effects for socio-demographics, time, intervention, time-by-intervention interaction, and random effects for individual educators nested within services. Factor analysis and latent class analysis will be considered for secondary outcome items prior to being similarly analysed using generalised linear mixed effects models. Open-ended, qualitative data responses to process outcome items in the post-intervention director and educator surveys will be thematically analysed according to a prepared coding framework and consensus reached by at least two research team members not involved with data collection. For any discrepancies, a third team member will make the final decision. Other process evaluation data will be summarised descriptively. All quantitative data preparation and analyses will be carried out using SAS 9.4 or above and Stata 17 or above, and qualitative analysis using NVivo 20.0 or above.

As part of dissemination, a study report presenting the overall findings of the research will be prepared for all participants and stakeholders. Peer-reviewed publications and presentations will allow the results to be disseminated to the scientific community. Results will be reported according to Consolidated Standard of Reporting Trials guidelines [26].

## Discussion

The first 5 years of life are critical for establishing health behaviours such as physical activity which enables children to have both a healthy childhood and decreases their risk of later chronic disease. Most young children do not have enough opportunity to be active. ECEC is a key setting to intervene to increase physical activity in the early years [41]. Physical activity has not to date, been a focus for educators or the ECEC sector, and at best provides a ‘piecemeal’ approach to physical activity promotion in early childhood. This study rectifies this situation by providing evidence of the effectiveness of the Play Active program.

The Play Active program will enable ECEC services to provide supportive policy environments that give children in their care a physically active and healthy start to life. Play Active provides ECEC services with an evidence-informed physical activity policy intervention and policy implementation support strategies to improve educator physical activity practices and positively impact children’s physical activity levels whilst attending care. Considering a large proportion of children attend ECEC, Play Active has the potential to reach a significant number of young children. With the ECEC environment accounting for almost a half of the variation in children’s physical activity levels, findings from this study are critical for informing a scale-up and adaptation of the Play Active program to improve children’s physical activity levels, health and wellbeing.

## Supplementary Information

**Additional file 1.**

**Additional file 2.**

## Data Availability

A copy of the ECEC specific physical activity policy template is available from the corresponding author by email request. The de-identified datasets generated from this study will be available for analytic purposes by academics and research staff if approved by the Principal Investigator and relevant ethics committees, from 12 months following the anticipated project end date.

## Abbreviations

ECEC: Early Childhood Education and Care
NAPSACC: Nutrition and Physical Activity Self-Assessment for Child Care
EPAO: Environment and Policy Assessment and Observation
