# Supplementary material for: Evaluating the effectiveness of the Play Active policy intervention and implementation support in early childhood education and care: a pragmatic cluster randomised trial protocol
Source: BMC Public Health. 2022 Feb 14;22:306. doi: 10.1186/s12889-022-12729-5 (PMC8842565; doi:10.1186/s12889-022-12729-5)
Supplement: Supplementary file 2 — Additional file 2. [file 12889_2022_12729_MOESM2_ESM.docx]

**Additional file 2 -** Itemised Play Active early childhood education and care (ECEC) physical activity policy implementation support strategies

| **Implementation support strategy** | **Itemised components** |
| --- | --- |
| 1. **Personalise policy** | 1. ECEC services are encouraged to **tailor** the physical activity policy template to their service and asked to do this within the first 2 months of receiving the policy template. This serves to enhance **policy** **ownership**. This also aligns with the program being an **adaptable** intervention |
|  | 1. To assist ECEC services to tailor their physical activity policy they are asked to include at least **five** practices (from the 25 in the policy template) into their own physical activity policy^1^ |
|  | 1. **Seven** **prioritised** high-impact and low-effort practices are recommended to services. They are: 2. ensuring awareness and understanding of the physical activity policy; 3. providing many daily opportunities for outdoor playtime; 4. providing opportunities for children to engage in discovery learning and discussing the importance of physical activity 5. breaking up prolonged periods of sedentary behaviour (e.g. sitting or standing for long periods or confining infants to high chairs or cots when not eating or sleeping); 6. not using punitive measures such as withholding physical activity as punishment for managing challenging behaviours (e.g. seated time out) and not using physical activity as punishment (e.g. star jumps); 7. wearing comfortable and appropriate clothing and footwear that doesn’t limit children’s and educator’s ability to engage in physical activity; 8. making the physical activity policy available to staff, families and visitors. |
| 1. **Policy review and approval** | 1. Policy **audit** and **feedback** – after submission of the draft policy by the service, the policy is reviewed by two Project Officers and tailored feedback is provided within one week of submission if the draft policy doesn’t meet the required standards^2^ |
|  | 1. If the policy meets the required standards^2^, **approval** is given for the policy and the remaining implementation support strategies are provided |
|  | 1. After approval**,** a **poster** is sent to each service that includes their selected practices (from five to 25 practices, depending on what they selected (see 1.2)) |
| 1. **Resource guide** | - 1. An 89-page resource guide is provided as a **hard copy (bound) and digital PDF** version to services |
|  | - 1. A QR code within the document links to a ‘**link tree’** of digital resources, including: a 2-minute Play Active video, a 20-minute Play Active educator webinar, expression of interest link for Play Active and links to all of the digital resources mentioned in the resource guide (48 total) |
|  | - 1. The resource guide is **mapped** to the 25 practices^1^ outlined in the policy, expanding on the content in the policy template, and acting as a specific reference for each of the 25 practices |
|  | - 1. The guide is **consistently structured** into three sections for each procedure: (i) What can you do; (ii) What does it mean; (iii) Helpful resources |
|  | - 1. **Credibility** features are included in the resource guide (e.g. government and non-government partner endorsement, Minister for Community Services foreword) |
|  | - 1. Links made to ECEC National Quality Standards (where relevant) throughout the guide – **policy alignment** |
| 1. **Brief assessment tool** | - 1. **Practice 9** (of 25 practices in the physical activity policy) is the Energetic Play Assessment Tool. Within four standardised periods of the day (arrival to morning tea, to lunch, to afternoon tea, to departure) the degree to which each child engages in energetic play is scored (five point scale: very rarely energetic to very often energetic) by educators. |
| 1. **Professional development** | - 1. **Practice 12** (of the 25 practices in the physical activity policy) outlines educators taking part in professional development to increase knowledge and skills around supporting children’s physical activity |
|  | - 1. KIDDO evidence-based online training: Six 30-60 minute modules including resources, training, quizzes and assessment. Content is focused on developing educators skills to develop students fundamental movement skills. Access to the training is free for trial services and made available to educators via email. |
|  | - 1. Nature Play WA evidence-based online training: Five modules, each approximately 3-4 hours long, to be completed at service level over 4-6 weeks. Content is focused on the importance of being a playful educator; active and playful outdoor learning environments; using outdoor play to increase physical activity; planning cycles for active outdoor play; and documenting as a process of reflection, intention and communication. Educators encouraged to complete this training through the resource guide and the Project Officer. The training has a cost of AUD$49 per module, total cost AUD$686. |
|  | - 1. Nature Play WA evidence-based face to face training: Services are offered a 2-hour face to face training course, at a cost of AUD$550. Content is an abridged version of the Nature Play WA online training. |
| 1. **Project Officer implementation support** | - 1. Weekly phone call and/or email to remind services to submit their draft policy for review and approval. |
|  | - 1. A mid-implementation (6 weeks) check-in phone call made by the Project Officer to the services to determine whether policy implementation has commenced. |

^1^The 25 practices are expanded to 27 practices in the policy template, as two additional practices are added which are also implementation support strategies (policy review and approval; the resource guide).

^2^ The minimum requirements for physical activity policies to be approved are that it includes: two (out of two) key statements; nine (out of nine) physical activity and sedentary behaviour recommendations; and at least five (out of 25) practices to prioritise implementing during the trial’s intervention period.
